# Items

**Published:** 1898-02

**Authors:** 


### Items.

THE sixth annual report of the Sheppard Asylum, a hospital for mental diseases, located at Towson, Maryland, is a handsome and well-illustrated brochure. Interest has increased in this institution since Mr. Enoch Pratt's bequest, a gift that will double the capital and increase the scope and usefulness of the institution. The trustees have wisely accepted the trust and all that remains is for the legislature to perform its part when this will become one of the largest, if not the most useful private hospital for the insane in the country. Dr. Edward N. Brush is the superintendent.

---

The fifth annual report of the Children's Hospital at Buffalo is an interesting little pamphlet. In it we read that Dr. Schuyler Wheeler, of New York, gave the hospital a $5,000 endowment in memory of his little son Richard. In addition Dr. Wheeler has fitted up one of the small rooms in a most complete mnnner, making it attractive with dainty furnishings in blue and white, and in which a memorial window is to be placed, made by Tiffany. The nurse's home, attached to the hospital, has been freed from debt by the payment during the year of $2,000. Taken altogether this little hospital is doing most creditable work and is a charity that appeals to the sympathy of every human heart.

---

Dr. G. A. Stockwell, formerly employed by Parke, Davis & Company, Detroit, has entered the service of Henry K. Wampole & Company, Philadelphia. Dr. Stockwell announces that he will probably soon issue a new high-class and wholly independent medical journal that will be entirely separate from his connection with the Philadelphia house.

---

Mathews's *Quarterly Journal of Rectal and Gastro-Intestinal Diseases* announces that it will conduct a special train from St. Louis to Denver for the accommodation of persons who desire to attend the meeting of the American Medical Association in June, 1898. Further information regarding the proposed train can be had by addressing the Quarterly special, P. O. Box 434, Louisville, Ky.

---

The Chutmuck Special is the name of a train that the Missouri Pacific Railway has arranged to run from St. Louis to Denver for the meeting of the American Medical Association in June, '98, Information may be obtained by addressing Col. H. C. Townsend, general passenger and ticket agent, St. Louis, Mo.

---

The second annual report of the managers of the Second Hospital for the Insane of the State of Maryland for the year ending October 1, 1897, is received. The construction of new buildings is proceeding, the first group being finished. It consists of a service building and three cottages arranged on the crest of a knoll surrounding a large open space. The location on the old Patterson farm at Sykesville is as near ideal as can be. About fifty patients have been treated during the past year and many more will be provided for as the buildings progress. Dr. George H. Rohé is the superintendent.

---

THE Clyde Line fleet of steamships plying between New York, Charleston, S. C., and Jacksonville, Florida, are, without doubt, the smoothest sailing vessels between the North and South. Built by expert ship-builders, regardless of cost, they are the safest and most comfortable steamers in the coastwise trade. The staterooms are commodious, airy and elegantly furnished.

The tables on the various steamers are furnished with all the delicacies that the most fastidious can expect. When one takes into consideration that both the Northern and Southern markets are at the command of the Clyde Line, it is no wonder that they have the reputation for furnishing the best meals that money can buy.

---

AN INTERNATIONAL health exposition will be held at Grand Central Palace, New York city, for five weeks beginning April 25, 1898. This will be an exhibit of foods, sanitary appliances, systems of house heating, ventilation and plumbing, hospital arrangements, systems of sewerage, model school rooms, model tenement houses, electric apparatus and the like.

The list of the advisory board already appointed comprises more than ninety specialties in the various lines of sanitary work, among whom may be mentioned: Dr. Stephen Smith, president department public charities, New York; Hon. Andrew H. Green; Albert Shaw, editor *Review of Reviews*; Edward Atkinson, Alfred T. White, ex-commissioner of public works, city of Brooklyn; Samuel Gompers, president American federation of labor; Miss Frances E. Willard, president W. C. T. U.; Dr. H. W. Wiley and B. G. Fernow of the U. S. department of agriculture; Mrs. Melville Dewey; Col. Wm. G. Church, editor of the *Army and Navy Journal*; Dr. H. P. Baker, secretary Michigan state board of health; Dr. Mary Putnam Jacobi; Dr. George F. Shrady, editor *Medical Record*.

